# Assessing the impact of vaccines on COVID-19 efficacy in survival rates: a survival analysis approach for clinical decision support

**DOI:** 10.3389/fpubh.2024.1437388

**Published:** 2024-11-18

**Authors:** Juan Luis González Rodríguez, Andreea M. Oprescu, Sergio Muñoz Lezcano, Jaime Cordero Ramos, Juan Luis Romero Cabrera, Miguel Ángel Armengol de la Hoz, Ángel Estella

**Affiliations:** ^1^Big Data Department, Andalusian Public Foundation Progress and Health-FPS, Seville, Spain; ^2^Electronic Technology Department, Universidad de Sevilla, Sevilla, Spain; ^3^Research Institute for Innovation & Technology in Education (iTED), Universidad Internacional de La Rioja (UNIR), Logroño, Spain; ^4^Hospital Pharmacy Central Management, Extremadura Health Service, Badajoz, Spain; ^5^Pharmacotherapeutic Monitoring and Personalized Medicine Group (IBIS-DS-25), Institute for Biomedical Research in Sevilla (IBIS), Sevilla, Spain; ^6^Lipids and Atherosclerosis Unit, Maimonides Institute for Biomedical Research in Cordoba (IMIBIC), Reina Sofia University Hospital, University of Córdoba, Córdoba, Spain; ^7^CIBER in Physiopathology of Obesity and Nutrition (CIBEROBN), Instituto de Salud Carlos III, Madrid, Spain; ^8^Intensive Care Unit University Hospital of Jerez, Medicine Department University of Cádiz, INIBiCA, Cádiz, Spain

**Keywords:** vaccine, COVID-19, clinical decision-making, predictive modeling, clinical decision support system

## Abstract

**Background:**

The global COVID-19 pandemic, caused by the SARS-CoV-2 virus, has presented significant challenges to healthcare systems worldwide.

**Objective:**

This study, based on an analysis of a cohort from the Public Health System of Andalusia (Spain), aims to evaluate how vaccination affects case-fatality rate in patients hospitalized due to COVID-19 infection in Andalusia.

**Methods:**

The cohort consists of 37,274 individuals after applying the inclusion criteria. We conducted survival analyses employing the Cox proportional hazards models and generated adjusted survival curves to examine the outcomes. The analyses were performed from three perspectives: vaccinated vs. unvaccinated patients, vaccinated and unvaccinated patients grouped by age, and stratified by vaccination status.

**Results:**

Results indicate a substantial correlation between vaccination and a 20% reduction in the risk of case-fatality. Age-specific effects reveal varying degrees of protection across different age groups.

**Conclusion:**

These findings emphasize the pivotal role of vaccination status in COVID-19 risk assessment, supporting the development of a clinical decision support system for accurate predictions and optimizing healthcare management at admission.

## 1 Introduction

The global COVID-19 pandemic, caused by the SARS-CoV-2 virus, has resulted in an unprecedented healthcare crisis since early 2020, exerting immense pressure on healthcare systems worldwide (1, 2). The high incidence and mortality rates of the disease have critically depleted healthcare resources globally, necessitating extraordinary measures such as resource rationing (3, 4). Amidst uncertainties surrounding disease behavior, clinical management, and resource allocation, the scientific community has been vigorously engaged in extensive research efforts.

Despite significant progress in understanding the disease—including genome sequencing (5), drug utilization (6–8), and vaccine development (9)—there remains a notable absence of robust prognostic tools for predicting severe outcomes or short-to-medium-term mortality. This highlights the urgent need for tools and systems that support clinical decision-making and resource allocation based on the prediction of disease progression toward severe forms, ICU admission, and the need for mechanical or non-invasive ventilation, along with short-to-medium-term mortality. Previous studies have validated predictive scales for clinical deterioration and mortality in similar clinical scenarios using vital signs and clinical variables upon admission. Notable examples include the Sequential Organ Failure Assessment (SOFA) for sepsis patients (10, 11), the Acute Physiology and Chronic Health Evaluation II (APACHE II) scale for ICU patients (12), and the National Early Warning Score 2 (NEWS2) scale used in the British healthcare system. Some of these scales have demonstrated acceptable predictive results for severe outcomes and mortality in COVID-19 patients (13).

Identified risk factors for clinical deterioration and short-to-medium-term mortality in COVID-19 include advanced age, pre-existing conditions (such as hypertension, diabetes, obesity, cardiovascular diseases, immunosuppression, and immunodepression), analytical parameters (such as troponin, D-dimer, lymphocytes, and ferritin), patterns in chest X-rays upon admission, vital signs in the first 24 h, and the development of complications [such as acute respiratory distress syndrome (ARDS) and myocardial involvement] (14, 15).

Recent studies, such as those by Escobar et al., have employed machine learning for predictive modeling and alerts for high-risk clinical deterioration in hospitalized patients, facilitating rapid responses and improved resource management, ultimately leading to a reduction in mortality (16). These analyses and predictive models have shown promising results in populations affected by COVID-19 (17–19). Additionally, Waku et al.'s (20) research examines disease dynamics and forecasts transitions between epidemic and endemic phases of COVID-19, and assesses the impact of vaccination.

Developing a clinical decision support system based on predictive models for short-to-medium-term case-fatality rates in COVID-19 patients, utilizing patient clinical variables, laboratory parameters, and imaging, holds significant potential for improving resource management and patient care, potentially reducing mortality. Our study seeks to create a comprehensive predictive tool for effective risk assessment of COVID-19 patients. This will be accomplished by incorporating demographic information, vaccination status, healthcare setting, clinical outcomes, pandemic waves, pre-existing conditions, and laboratory parameters into the development of multivariate proportional hazards models, thereby providing a thorough analysis of the impact of vaccines on case-fatality rates among COVID-19 patients.

In this regard, it is important to note that the vaccines used in Andalusia, their timelines of introduction, and potential factors influencing their efficacy play a significant role in the observed case-fatality rates. The Pfizer vaccine was introduced at the end of 2020, followed by Moderna in March 2021. AstraZeneca began its rollout in June 2021, and Janssen, the single-dose vaccine, was introduced shortly after in mid-2021. The variation in vaccine distribution and administration, influenced by factors such as evolving public health recommendations, the emergence of new virus variants, and regional capacity for distribution, has been integrated into our analysis. This provides a comprehensive understanding of the impact of these vaccines on COVID-19 mortality across different waves of the pandemic.

The development process of the clinical decision support system involves three main phases. Initially, the process includes the request, pseudonymization, and processing of data, followed by the selection, training, and hyperparameter tuning of the model, and culminating in the evaluation of prediction metrics. If the metrics of this retrospective model prove promising, we will advance to the subsequent phase. The second phase entails implementing the system in pilot hospitals, conducting data download tests, correcting software and operational errors, and assessing initial results for potential clinical benefits. Ultimately, if the results are favorable, a future phase will proceed with the prospective real-time development and validation, integrating the system into the Electronic Health Record and connecting it to various data sources, while ensuring privacy and obtaining informed consent from patients. Our study focuses on the initial phase.

## 2 Materials and methods

The study cohort consisted of COVID-19 patients from the Andalusian Public Health System (Sistema Sanitario Público Andaluz, SSPA) who were admitted to participating hospitals. The dataset initially included 59,001 admissions, all of whom were 18 years old or older. This temporal structure provides a cohort defined according to specific pandemic periods: Wave 1 (01/01/2020–10/05/2020), Wave 2 (11/05/2020–20/12/2020), Wave 3 (21/12/2020–07/03/2021), Wave 4 (08/03/2021–20/06/2021), Wave 5 (21/06/2021–10/10/2021), Wave 6 (11/10/2021–27/03/2022), and Wave 7 (28/03/2022–31/12/2022) (21). This temporal structure offers a comprehensive overview of hospital admissions across various phases of the pandemic.

The analysis of vaccine consumption in Andalusia during the different pandemic waves reveals the evolution of the administration of the four main vaccines: Pfizer developed its mRNA-based vaccine, Comirnaty, while Moderna produced its own mRNA vaccine, branded as Spikevax. AstraZeneca introduced Vaxzevria, a viral vector vaccine, and Janssen, marketed as Jcovden, provided a single-dose viral vector option (22).

During Wave 3 (March 7, 2021), the Pfizer vaccine had already reached a total of 743,428 doses administered. Meanwhile, the Moderna vaccine was still in its initial phase, with 39,914 doses administered. At this point, no doses of AstraZeneca or Janssen had been administered. In Wave 4 (June 21, 2021), there was a significant increase in the administration of all vaccines. Pfizer doses rose to 4,707,383, while Moderna reached 574,251 doses. AstraZeneca had started to be administered, with 1,117,484 doses administered, and Janssen was introduced with 116,562 doses. By Wave 5 (October 20, 2021), Pfizer vaccine administration continued to grow, reaching 9,098,680 doses. Moderna doses also increased, reaching 1,723,404, while AstraZeneca recorded 1,758,232 doses. The Janssen vaccine reached 319,344 doses administered by this point. In Wave 6 (March 29, 2022), Pfizer continued its increase, reaching 14,304,189 doses. Moderna nearly doubled its total compared to the previous wave, reaching 3,557,961 doses administered. AstraZeneca recorded 1,784,771 doses, and Janssen reached 327,141 doses. Finally, in Wave 7 (January 3, 2023), Pfizer maintained its total of 14,304,189 doses without further increases from the previous wave. Moderna administration rose slightly to 3,663,365 doses, while AstraZeneca reached 1,789,825 doses. The administration of Janssen stabilized at 328,326 doses. This progression illustrates the varied adoption of each vaccine throughout the waves, with Pfizer remaining the dominant vaccine in terms of total doses administered, followed by Moderna, AstraZeneca, and Janssen.

**Figure 1:**
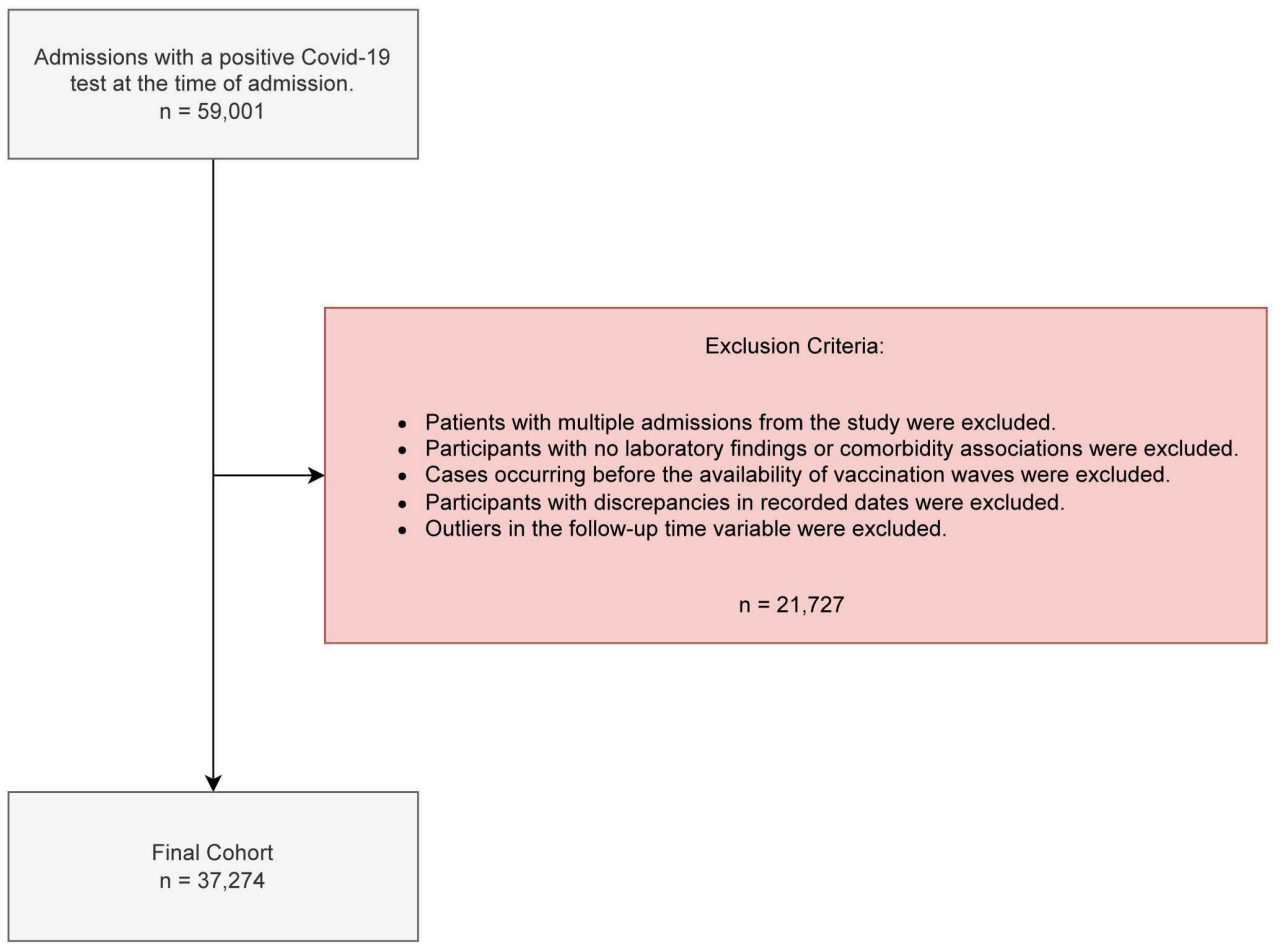
Criteria for inclusion/exclusion and reasons for data removal.

The following exclusion criteria were applied, as illustrated in Figure 1. First, for patients with multiple admissions, we retained only the last admission, as it is the only one where fatality could occur. In the context of COVID-19 in Andalusia, the case-fatality rate is defined as the proportion of deaths among identified confirmed cases of the disease. Specifically, it measures the severity of the disease by dividing the number of deaths attributed to COVID-19 by the total number of confirmed cases, expressed as a percentage. This metric is distinct from mortality, which refers to the incidence of death within the general population over a specific period, providing insights into the overall impact of the disease on the regional population without directly linking deaths to confirmed cases. Consequently, 1,793 admissions were excluded. Second, 7,212 admissions corresponding to patients whose records lacked laboratory data or associated comorbidities in the Electronic Health Record were excluded. Additionally, admissions from Wave 1 (17 admissions) and Wave 2 (12,481 admissions) were excluded due to the absence of vaccination during these periods. Validation criteria were applied to address discrepancies between the admission, discharge, or expiration dates, resulting in the removal of 26 admissions.

We defined a follow-up time variable based on patient outcomes. If the patient died, the variable was defined as the number of days between the admission and expiration date. If the patient survived, the variable is calculated as the number of days elapsed between admission and discharge date plus 30 days, reflecting that the patient remained alive for at least 30 days post-discharge. Upon examining this follow-up time, we observed that its distribution was right-skewed. We decided to eliminate admissions where follow-up time fell within the top 0.5% of the entire study population, resulting in 198 cases being excluded. To achieve this, we employed the winsorization technique, which involves capping or trimming extreme values of follow-up times to ensure that outliers do not disproportionately influence the analysis.

For records with laboratory data but missing variables, we used the Iterative Imputation technique, which involves iteratively modeling each feature with missing values as a function of other features. This approach uses a regressor to predict unavailable values for each feature through successive iterations. Variables where more than 50% of the patients had missing values were imputed. This process yielded a final cohort of 37,274 admissions, all of which were confirmed COVID-19 positive upon admission and available for analysis after applying the exclusion criteria.

Data were obtained following ethical approval from the Committee for Biomedical Research in Andalusia (Comité para la Investigación Biomédica en Andalucía, CCEIBA) through Protocol Version 1.4 dated August 27, 2021, and HIP/CI Version 1.0 dated August 27, 2021. The internal protocol code associated with the approval is 1303-N-21.

We conducted survival analyses with the following considerations: (i) a general model, with a cohort stratified into vaccinated and unvaccinated patients; (ii) a model by age groups, with the cohort stratified into vaccinated and unvaccinated patients, grouped by age; and (iii) a model stratified by immunization groups, with the cohort categorized by vaccination status: unprotected, incomplete vaccination, and complete vaccination. We assessed the relationship between survival outcomes and various factors, such as demographic information and clinical data. Specifically, the model incorporated variables such as age, clinical laboratory parameters, comorbidities, and date of admission to comprehensively evaluate potential predictors of survival. The model summary, including Hazard Ratios and confidence intervals, was generated to quantify the impact of each variable on survival. We used a survival analysis approach to investigate the impact of vaccination status on patient outcomes.

In this study, we defined the event as the case fatality rate among COVID-19 patients admitted to the hospital with a confirmed positive diagnosis upon admission. The event includes patients who die during hospitalization as well as those who die within 30 days following discharge. For patients without an expiration date in the dataset, the survival time is unknown. The following rationales have been considered: (a) patients who are hospitalized beyond the data collection period; (b) patients lost to follow-up, resulting in a lack of information during the study; (c) patients facing alternative events hindering continuous follow-up, such as traffic accidents. We assumed that, in all three cases, the patients had not died. The Hazard Ratio allows us to compare the risk of an event occurring between groups. It is expressed as the ratio of their instantaneous risk functions, enabling us to assess the influence of different risk factors on the event over time (23).

For the first analysis, patients were classified as “vaccinated” if they had received at least one vaccine dose at least 14 days prior to the hospital admission date. Conversely, patients who had not received any doses before hospitalization were considered “unvaccinated.”

For the second analysis, we divided the already stratified vaccinated and unvaccinated cohort (maintaining the definition from our first analysis) into different groups according to patient age. The defined age groups are as follows: Group A, ≥80 years; Group B, ≥70 years; Group C, ≥60 years; Group D, ≥40 years; Group E, ≥18 years. Each group corresponds to the starting date of the vaccination campaign periods in Spain: Group A, 27/12/2020; Group B, 05/04/2021; Group C, 03/05/2021; Group D, 01/06/2021; Group E, 20/07/2021. The analysis by age groups was designed to enhance our understanding of how vaccination impacts survival outcomes of patients of different ages, considering the timelines of the vaccination campaigns for each group.

For the third analysis, in which the cohort was stratified by vaccination status, we categorized the patients into three distinct subgroups: unprotected, incomplete, and complete. This cohort corresponds to the period in which the entire population had access to vaccination. The unprotected group encompassed unvaccinated patients who had not received any vaccine dose. The incomplete group comprised patients who had received at least one dose but fewer than three, with the last dose administered more than 180 days prior. Lastly, the protected group included patients who had received a minimum of three vaccine doses, with the time interval between the last dose and admission falling within 180 days, indicating ongoing protection.

For data cleaning and preprocessing, Python and Jupyter notebooks were utilized to facilitate a comprehensive understanding of the statistical procedures applied. The Scikit-learn library for Python was used to impute missing values. The survival analysis was executed in R, using Rmarkdown notebooks for enhanced reproducibility.

## 3 Results

A case fatality rate of 7,202 (19.3%) was observed within the cohort, of which 3,476 (48.3%) had received at least one vaccine dose. The median age of patients who died was 81 years, in contrast to a median age of 64 years among survivors. Patients who died had received a median of 0 vaccine doses, whereas survivors had received a median of two doses. ICU stays were markedly elevated among the deceased, indicating a pronounced association between ICU admission and adverse outcomes. Patients who died had a median of 10 inpatient days compared to 5 days for survivors. Additionally, follow-up days were notably shorter for patients who died, with a median of 10 days compared to 37 days for survivors. The clinical and sociodemographic characteristics of the cohort are presented in Table 1.

**Table 1:** Study population characteristics.

**Variable**	**Level**	**Stratified by case-fatality rate**				
		**Missing**	**Overall**	**Deceased**	**Survivor**	***p*** **-Value**
*n*			37,274	7,202	30,072	
Sex, *n* (%)	Female	0	2,1420 (57.5)	4,211 (58.5)	17,209 (57.2)	0.057
	Male		1,5854 (42.5)	2,991 (41.5)	12,863 (42.8)	
Age, median [Q1–Q3]		0	68 [54–80]	81 [72–87]	64 [51–77]	< 0.001
Vaccine doses administered, median [Q1–Q3]		0	0 [0–2]	0 [0–3]	0 [0–2]	< 0.001
Medical center type, *n* (%)	County hospital	0	8,445 (22.7)	1,940 (26.9)	6,505 (21.6)	< 0.001
	Regional hospital		14,888 (39.9)	2,634 (36.6)	12,254 (40.7)	
	Specialized hospital		13,941 (37.4)	2,628 (36.5)	11,313 (37.6)	
Vaccination status, *n* (%)	Yes		14,550 (39.0)	3,476 (48.3)	11,074 (36.8)	
ICU stay, *n* (%)	Yes	0	3,435 (9.2)	1,490 (20.7)	1,945 (6.5)	< 0.001
Inpatient days, median [Q1–Q3]		0	8 [5–13]	10 [5–20]	7 [5–12]	< 0.001
Followup_days, median [Q1–Q3]		0	36 [33–41]	10 [5–20]	37 [35–42]	< 0.001
Wave 3, *n* (%)	Yes	0	11,873 (31.9)	2,700 (37.5)	9,173 (30.5)	< 0.001
Wave 4, *n* (%)	Yes	0	7,053 (18.9)	648 (9.0)	6,405 (21.3)	< 0.001
Wave 5, *n* (%)	Yes	0	5,090 (13.7)	817 (11.3)	4,273 (14.2)	< 0.001
Wave 6, *n* (%)	Yes	0	6,951 (18.6)	1,628 (22.6)	5,323 (17.7)	< 0.001
Wave 7, *n* (%)	Yes	0	6,307 (16.9)	1,409 (19.6)	4,898 (16.3)	< 0.001
Pre-existing condition active cancer, *n* (%)	Yes	0	5,415 (14.5)	1,529 (21.2)	3,886 (12.9)	< 0.001
Pre-existing condition asthma, *n* (%)	Yes	0	5,002 (13.4)	721 (10.0)	4,281 (14.2)	< 0.001
Pre-existing condition congestive heart failure, *n* (%)	Yes	0	8,693 (23.3)	2,588 (35.9)	6,105 (20.3)	< 0.001
Pre-existing condition chronic liver, *n* (%)	Yes	0	3,477 (9.3)	643 (8.9)	2,834 (9.4)	0.201
Pre-existing condition chronic kidney disease, *n* (%)	Yes	0	6,367 (17.1)	1,906 (26.5)	4,461 (14.8)	< 0.001
Pre-existing condition COPD, *n* (%)	Yes	0	7,943 (21.3)	2,023 (28.1)	5,920 (19.7)	< 0.001
Pre-existing condition dementia, *n* (%)	Yes	0	6,556 (17.6)	2,059 (28.6)	4,497 (15.0)	< 0.001
Pre-existing condition diabetes, *n* (%)	Yes	0	13,313 (35.7)	3,249 (45.1)	10,064 (33.5)	< 0.001
Preexisting condition hyperlipidemia, *n* (%)	Yes	0	20,295 (54.4)	4,236 (58.8)	16,059 (53.4)	< 0.001
Pre-existing condition hypertension, *n* (%)	Yes	0	24,948 (66.9)	5,955 (82.7)	18,993 (63.2)	< 0.001
Pre-existing condition ischemic heart disease, *n* (%)	Yes	0	4,592 (12.3)	1,316 (18.3)	3,276 (10.9)	< 0.001
Pre-existing condition obesity, *n* (%)	Yes	0	9,362 (25.1)	1,557 (21.6)	7,805 (26.0)	< 0.001
Pre-existing condition stroke, *n* (%)	Yes	0	1,994 (5.3)	546 (7.6)	1,448 (4.8)	< 0.001
Alanine transaminase (U/L), median [Q1–Q3]		0	31.0 [19.0–42.9]	27.0 [16.8–40.0]	31.6 [20.0–44.0]	< 0.001
Aspartate transaminase (U/L), median [Q1–Q3]		0	37.2 [25.0–50.0]	40.5 [25.8–55.0]	37.0 [24.4–48.2]	< 0.001
Creatinine (mg/dL), median [Q1–Q3]		0	1.0 [0.8–1.2]	1.2 [0.9–1.8]	1.0 [0.8–1.2]	< 0.001
C-reactive protein (mg/L), median [Q1–Q3]		0	89.2 [39.9–134.0]	107.8 [64.5–175.0]	82.2 [35.8–123.0]	< 0.001
D-dimer (ng/mL), median [Q1–Q3]		0	1,088.0 [561.0–2,272.2]	1,919.8 [940.0–3,670.0]	960.0 [519.0–2,063.2]	< 0.001
Glucose (mg/dL), median [Q1–Q3]		0	123.0 [103.0–149.0]	139.2 [112.0–178.0]	121.0 [103.0–143.0]	< 0.001
Hematocrit (%), median [Q1–Q3]		0	40.4 [37.0–44.2]	39.0 [34.0–43.1]	40.8 [37.6–44.4]	< 0.001
Hemoglobin (g/dL), median [Q1–Q3]		0	13.6 [12.4–14.9]	13.0 [11.3–14.2]	13.7 [12.7–15.0]	< 0.001
Normalized prothrombin time–INR (–), median [Q1–Q3]		0	1.1 [1.0–1.2]	1.2 [1.1–1.4]	1.1 [1.0–1.2]	< 0.001
Lactate Dehydrogenase (U/L), median [Q1–Q3]		0	338.0 [261.0–405.0]	366.9 [289.0–483.0]	329.0 [256.9–391.0]	< 0.001
Leukocyte count (× 10^3^/μL), median [Q1–Q3]		0	7.4 [5.3–9.4]	8.2 [6.0–11.7]	7.1 [5.2–8.9]	< 0.001
Lymphocyte count (× 10^3^/μL), median [Q1–Q3]		0	1.0 [0.7–1.4]	0.8 [0.5–1.3]	1.1 [0.7–1.4]	< 0.001
Lymphocyte percentage (%), median [Q1–Q3]		0	15.8 [9.6–20.3]	11.2 [6.4–16.6]	16.3 [10.7–21.1]	< 0.001
Mean corpuscular hemoglobin (pg), median [Q1–Q3]		0	29.6 [28.5–30.9]	29.7 [28.3–31.3]	29.6 [28.6–30.8]	< 0.001
Mean corpuscular volume (fL), median [Q1–Q3]		0	89.4 [86.1–92.9]	90.8 [87.3–95.4]	89.4 [85.9–92.3]	< 0.001
Neutrophil count (× 10^3^/μL), median [Q1–Q3]		0	5.7 [3.8–7.5]	6.4 [4.6–9.8]	5.4 [3.6–7.0]	< 0.001
Neutrophil Percentage (%), median [Q1–Q3]		0	75.7 [70.2–83.5]	81.2 [75.0–87.8]	75.5 [69.4–82.1]	< 0.001
Platelet Count (× 10^3^/μL), median [Q1–Q3]		0	210.0 [160.0–253.0]	202.0 [148.0–254.0]	211.9 [162.0–253.0]	< 0.001
Potassium (mEq/L in HM, mmol/L in other units), median [Q1–Q3]		0	4.2 [3.9–4.5]	4.3 [4.0–4.7]	4.1 [3.8–4.4]	< 0.001
Red blood cell count (× 10^6^/μL), median [Q1–Q3]		0	4.6 [4.2–5.0]	4.4 [3.8–4.8]	4.6 [4.3–5.1]	< 0.001
Sodium (mEq/L in HM, mmol/L in other units), median [Q1–Q3]		0	137.0 [135.0–139.0]	137.0 [134.0–140.0]	137.0 [135.0–139.0]	< 0.001
Urea (mg/dL), median [Q1–Q3]		0	41.0 [29.7–58.0]	61.0 [45.0–95.0]	38.0 [28.0–51.6]	< 0.001

**Figure 2:**
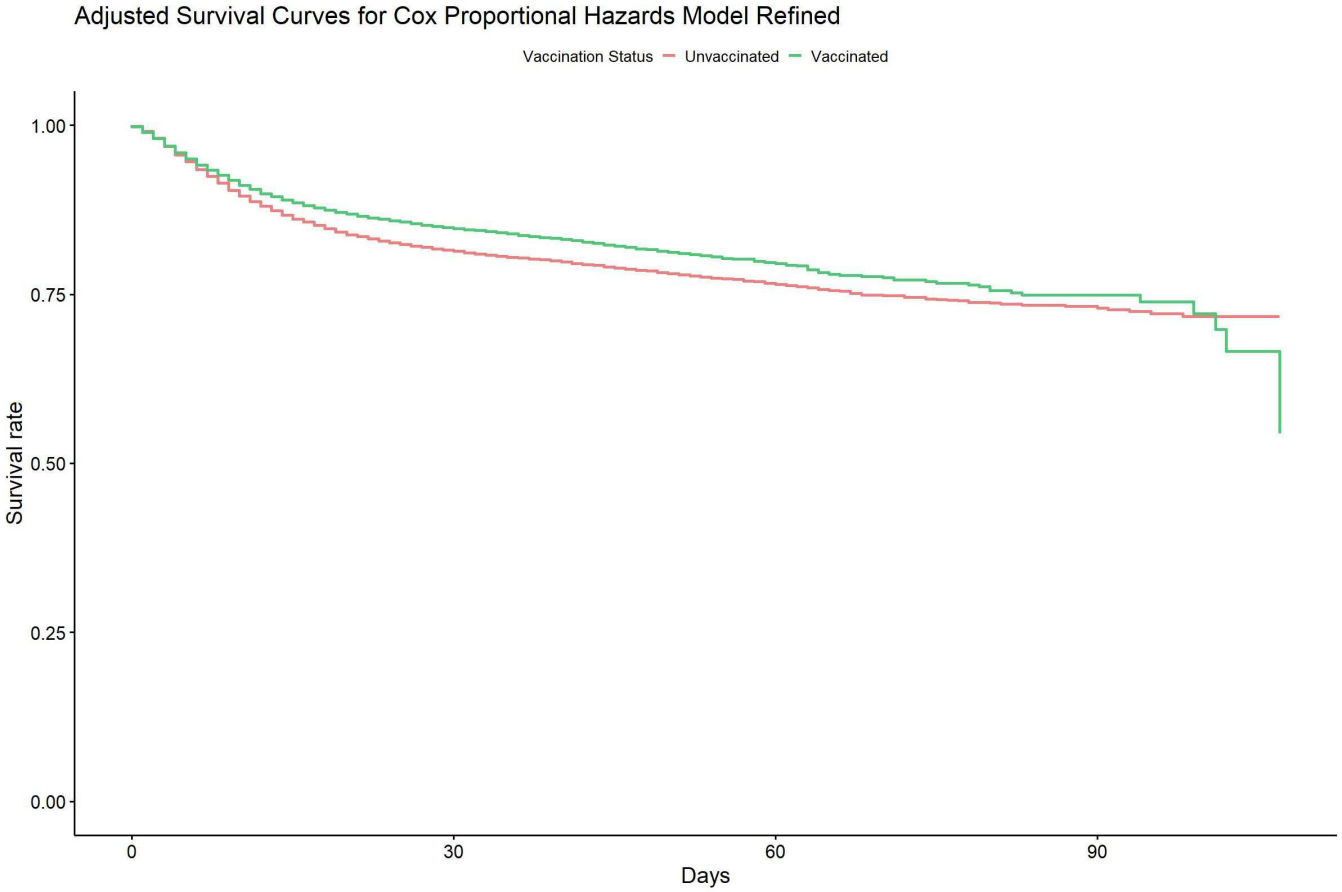
Adjusted survival analysis for Cox proportional hazards general model for case-mortality rate between unvaccinated and vaccinated population.

The Cox Proportional Hazards survival curve reveals a lower survival rate among unvaccinated patients (see Figure 2). The number of patients at risk at each stage of the analysis is available in Supplementary Figure 1.

**Figure 3:**
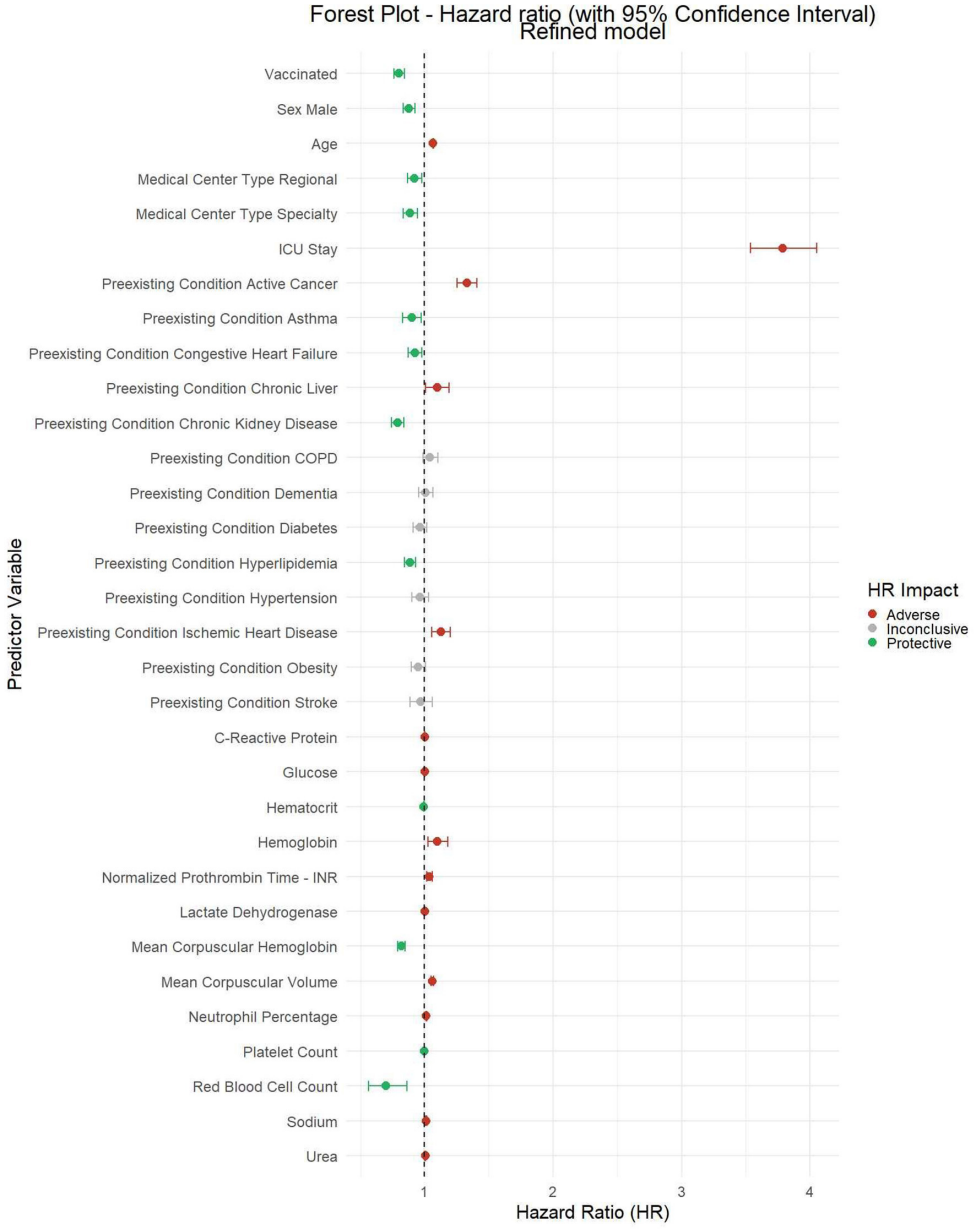
Forest plot of hazard ratios for general model for case-mortality rate between unvaccinated and vaccinated population.

We present the following results from the first analysis (general model). The “Vaccinated” variable, representing patients who received at least one vaccine dose at least 14 days prior to hospital admission, indicates a protective effect, with a Hazard Ratio of 0.80 (95% CI: 0.76–0.84; *p* = 0.026; see Figure 3). This finding underscores a correlation between vaccination and an immediate 20% reduction in the risk of case fatality rate compared to the unvaccinated group. Hazard ratios and confidence intervals for the general model addressing case-mortality rates between the unvaccinated and vaccinated populations are available in Supplementary Table 1.

Patients who died had a notably higher median age [81 (72, 87)] compared to those who survived [64 (51, 77)], suggesting that age is a key factor influencing case-fatality rate outcomes. Patients requiring ICU admission also exhibited a substantially higher case fatality rate. Pre-existing conditions, such as active cancer and ischemic heart disease, show strong correlations with higher case fatality rates. Additionally, C-Reactive Protein (CRP) levels present a significant difference between patients who died and those who survived (*p* < 0.001). Patients who died had a notably higher median CRP level [108.9 (66.2, 176.2)] compared to survivors [83.7 (37.1, 124.0)].

Finally, the median follow-up days variable significantly differed between deceased and surviving groups (*p* < 0.001), indicating that patients who died had a much shorter median follow-up period [10 (5, 20)] compared to survivors [38 (35, 42)].

**Figure 4:**
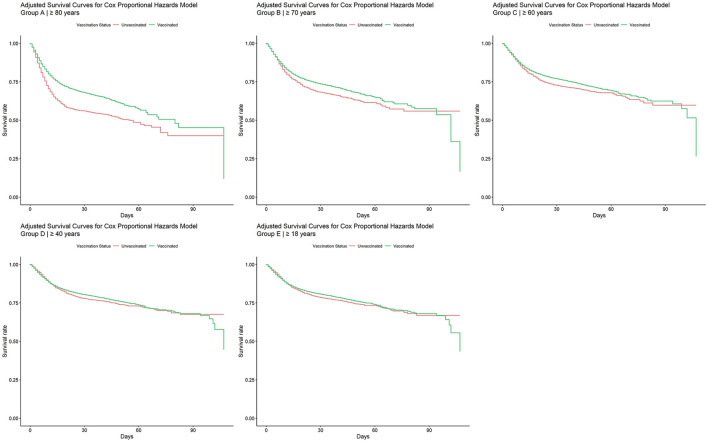
Adjusted survival curves for Cox proportional hazards model by age groups.

The second analysis (model by age groups) reveals that the protective effect of vaccination is more pronounced in patients aged 80 years and older (Group A), gradually diminishing across younger age groups (see Figure 4). In the 60-year-old group (Group B), the survival pattern shows less pronounced protection, and this trend continues to decrease in the 40-year-old group (Group C). Finally, in the 18 years and older group, the survival curves between vaccinated and unvaccinated patients intertwine, indicating a less marked difference in terms of protection. During the initial 25–50 days, a distinct divergence in survival outcomes is evident between patients who are unprotected and those who have some form of protection (see Supplementary Figure 5). Hazard ratios and confidence intervals for models by age groups addressing case-mortality rates between unvaccinated and vaccinated populations are available in Supplementary Tables 2A–E. Forest plots with the HR represented visually are available in Supplementary Figures 2A–E.

The third analysis (model stratified by immunization status) indicates a significant decrease in the risk of mortality for the incompletely vaccinated group (HR: 0.33, 95% CI: 0.30–0.36) compared to the fully protected group (HR: 0.30, 95% CI: 0.28–0.32), as shown in Supplementary Figure 6. Beyond the 50-day threshold, the trend between incompletely vaccinated and unvaccinated patients remains consistent, although with a less pronounced reduction in survival differences.

On the other hand, ICU stay is associated with a higher risk of mortality, with an HR of 2.03 (95% CI: 1.88–2.19), reflecting the clinical severity of these patients. Regarding age, the HR of 1.05 (95% CI: 1.05–1.05) shows that aging is associated with a slight increase in the risk of mortality. Similarly, lower hemoglobin levels are associated with an increased risk of mortality (HR: 1.11, 95% CI: 1.03–1.21).

Note that in Supplementary Figure 6, we exclusively represent the protected and incomplete groups, with the unprotected group serving as the reference line for hazard ratio (HR) calculation. Consequently, the unprotected group is not depicted in Supplementary Figure 6. Hazard ratios and confidence intervals for immunization-stratified groups addressing case-mortality rates between unvaccinated and vaccinated populations are available in Supplementary Table 3. The number of patients at risk in each stage of the analysis is provided in Supplementary Figure 7.

## 4 Discussion

This study places particular emphasis on discerning the influence of vaccination status on patient recovery rates and survival in hospitalized patients. The ultimate goal is to enhance the accuracy and interpretability of survival analysis methodologies, providing valuable insights into the impact of COVID-19 vaccines across different patient cohorts. The reduction in case-fatality rate observed in vaccinated patients pertains to a specific population—those who required hospitalization. Patients requiring hospital admission to medical wards or the ICU often present additional factors influencing their deterioration, primarily conditioned by their comorbidities in addition to the viral infection. Therefore, it is crucial to document the reduction in case-fatality rate that the vaccine provides despite the severity of the patient's condition. Consequently, the overall effect on reducing case-fatality rate, including hospitalizations prevented by the vaccine, is not considered in the percentage described in this population.

Our study reveals that demographic factors, particularly age, sex, and pre-existing conditions, significantly affect COVID-19 mortality. ICU stay was the most substantial predictor of death across all age groups, while vaccination consistently reduced mortality risk, reaffirming its importance in mitigating severe outcomes. Pre-existing conditions, particularly active cancer and chronic liver disease were associated with increased mortality risk, with older patients displaying a greater vulnerability to conditions such as COPD and diabetes. Additionally, males had a slightly lower risk of mortality compared to females, highlighting a modest yet significant impact of sex on outcomes.

The study aims to create a predictive tool in the form of a clinical prediction support system that incorporates various patient parameters for effective risk assessment. We document factors related to the prognosis of SARS-CoV-2 infection in patients who, despite vaccination, have required hospitalization. The study's design, based on Cox Proportional Hazard modeling, aims to provide decision support systems for clinical professionals to identify patients at risk of developing a complicated course of the disease.

Previous studies using similar methodologies did not consider the vaccination status of patients (24–26). Most studies identifying poor prognosis factors for SARS-CoV-2 infection were conducted before widespread vaccination, focusing on clinical risk profiles. In the Andalusian population admitted to the ICU, age, the development of organ failure, and severity upon ICU admission were related to case-fatality rate (27). This is crucial considering that many recommendations for managing SARS-CoV-2 infection are based on studies conducted before vaccination, raising questions about their applicability to patients with characteristics different from those in clinical trials. Vaccination effectiveness has been documented in numerous studies, showing a significant decrease in hospitalization (89.1%), ICU admission (97.1%), and case-fatality rate (97.3%) (28). In the U.S., it was documented that vaccination was around 90% effective in preventing hospitalizations and ICU admissions (29). Similar findings have been documented in Spain (30). Vaccination against SARS-CoV-2 has not only drastically decreased the number of hospitalizations and the development of more severe clinical forms of the infection but also seems to have influenced the decrease in other infections in hospitalized older adult patients (31).

Vaccination in Spain started with older adult patients and healthcare workers, progressively expanding to the remaining age groups. Despite being vaccinated, some patients still required hospital admission, possibly due to immune escape when complete vaccination schemes were not generated (32). Vaccine effectiveness in preventing new infections and severe forms of the disease has been well established (33). However, the impact of vaccination on case-fatality rate in hospitalized patients is not well-developed. Vaccinated patients who still require hospitalization may present a clinical profile that makes them more vulnerable to the infection, rendering the vaccine less effective. Factors such as cancer have been identified as related to worse prognosis, supporting studies showing reduced vaccine effectiveness in immunocompromised patients (34). Comorbidities such as chronic obstructive pulmonary disease have also been identified as factors associated with case-fatality rate (35). We also identified an increase in case-fatality rate in patients with a medical history of ischemic coronary artery disease, consistent with a systematic review that included 81 published studies involving 157,439 patients (36). Patients with higher levels of C-reactive protein (CRP) exhibited a worse prognosis.

The second analysis (model by age groups) confirms the protective effect of the vaccines even in patients requiring hospitalization. This protective effect of vaccination was more pronounced in older age groups, although the difference in survival from vaccination was less pronounced among younger vaccinated patients.

In the third analysis (model grouped by immunization status), we observed the protective effect of the vaccine when identifying three groups of patients: unprotected, incomplete (those who had exceeded 6 months after the last dose of the vaccine), and protected. The study confirms the protective effect of the vaccines even in hospitalized patients, considering vaccinated groups in different periods. The association between ICU admission and a worse prognosis is related to the fact that these admissions are intended for the most severe cases requiring invasive treatments.

This study has the following limitations. We did not measure antibody levels post-vaccination to assess efficacy, as it was a large population sample, and such determinations were not routinely performed in hospitalized patients. Concomitant medications were not considered, since the indications for antiviral treatment, monoclonal antibodies, and immunomodulatory treatments lacked universal indicators due to insufficient evidence and depended on the attending clinician's discretion. At certain times during the pandemic, treatments with questionable evidence were indicated due to the absence of well-designed studies to support them. We did not account for the different variants of the virus circulating during the various waves of the pandemic, as they were not determined in the majority of hospitalized patients.

Our study presents the following strengths. To date, this is the study with the largest sample of patients hospitalized for COVID-19 in which the effect of vaccination is analyzed using the Cox Proportional Hazards Model. Additionally, it considers not only the risk factors associated with mortality but also different age groups, and how the effect of the vaccine varies according to age.

Additionally, our study demonstrates that COVID-19 vaccination significantly reduces mortality risk across all age groups, with a consistent hazard ratio of 0.80. However, older populations, particularly those over 60, are at heightened risk due to age-related factors and pre-existing conditions such as chronic liver disease, COPD, and cancer, which further increase mortality. These findings suggest that routine COVID-19 vaccination should be prioritized for individuals over 60, with periodic booster doses considered to maintain immunity, similar to existing influenza vaccination strategies. For younger populations, vaccination should be tailored based on comorbidities and risk exposure, in addition to epidemiological reasons to prevent the spread of the disease and protect the most vulnerable population, aligned with the guidelines from health authorities. Overall, the study underscores the critical role of vaccination in reducing COVID-19 mortality and provides evidence to guide long-term vaccination policies, especially for high-risk groups.

This approach aims to reduce ICU admissions, long-term hospitalizations, and the overall strain on healthcare systems, leading to significant cost savings. Prioritizing vaccines for these groups, as well as healthcare workers, ensures effective use of resources and reduces the likelihood of severe outcomes and disruptions from future outbreaks.

The cost-benefit analysis shows that these policies can lower healthcare costs by preventing severe cases, reducing the burden on healthcare workers, minimizing long-term complications such as long COVID, and avoiding the economic consequences of lockdowns. By maintaining high immunity levels in high-risk populations, the strategy maximizes vaccine efficiency and reduces overall economic and healthcare burdens.

In conclusion, our study emphasizes the pivotal role of vaccination status in COVID-19 risk assessment. This highlights the importance of integrating vaccination status as a key factor, along with other demographic and medical data, in comprehensive risk assessment models. Based on these findings, there is a strong rationale for the development of a clinical decision support system, providing clinicians with a valuable tool to identify patients at higher risk and tailor interventions accordingly. From a policy-making standpoint, our results could aid managers and clinicians in refining admission protocols and treatment strategies.

## Data Availability

The datasets presented in this study can be found in online repositories. The names of the repository/repositories and accession number(s) can be found at: https://github.com/jlgonrod/survival_vaccination_analysis.
