# Supplementary material for: Assessing the impact of vaccines on COVID-19 efficacy in survival rates: a survival analysis approach for clinical decision support
Source: Front Public Health. 2024 Nov 18;12:1437388. doi: 10.3389/fpubh.2024.1437388 (PMC11609198; doi:10.3389/fpubh.2024.1437388)
Supplement: Supplementary file 1 [file Data_Sheet_1.docx]

Supplementary Material

# Supplementary Figures and Tables

For more information on Supplementary Material and for details on the different file types accepted. please see [here](https://www.frontiersin.org/guidelines/author-guidelines#supplementary-material).

## Supplementary Table 1

| **Cox Model Refined Report** | | | |
| --- | --- | --- | --- |
| **Variables** | **Hazard Ratio** | **Lower CI (95%)** | **Upper CI (95%)** |
| Vaccinated | 0.80 | 0.76 | 0.84 |
| Sex Male | 0.88 | 0.83 | 0.93 |
| Age | 1.07 | 1.06 | 1.07 |
| Medical Center Type Regional | 0.92 | 0.87 | 0.98 |
| Medical Center Type Specialty | 0.89 | 0.84 | 0.94 |
| ICU Stay | 3.78 | 3.53 | 4.05 |
| Preexisting Condition Active Cancer | 1.33 | 1.26 | 1.41 |
| Preexisting Condition Asthma | 0.90 | 0.83 | 0.97 |
| Preexisting Condition Congestive Heart Failure | 0.93 | 0.87 | 0.98 |
| Preexisting Condition Chronic Liver | 1.10 | 1.01 | 1.19 |
| Preexisting Condition Chronic Kidney Disease | 0.79 | 0.74 | 0.84 |
| Preexisting Condition COPD | 1.04 | 0.99 | 1.10 |
| Preexisting Condition Dementia | 1.01 | 0.95 | 1.07 |
| Preexisting Condition Diabetes | 0.96 | 0.91 | 1.02 |
| Preexisting Condition Hyperlipidemia | 0.89 | 0.84 | 0.93 |
| Preexisting Condition Hypertension | 0.97 | 0.90 | 1.03 |
| Preexisting Condition Ischemic Heart Disease | 1.13 | 1.06 | 1.20 |
| Preexisting Condition Obesity | 0.95 | 0.89 | 1.01 |
| Preexisting Condition Stroke | 0.97 | 0.89 | 1.06 |
| C-Reactive Protein | 1.00 | 1.00 | 1.00 |
| Glucose | 1.00 | 1.00 | 1.00 |
| Hematocrit | 0.99 | 0.99 | 1.00 |
| Hemoglobin | 1.10 | 1.03 | 1.18 |
| Normalized Prothrombin Time - INR | 1.04 | 1.02 | 1.06 |
| Lactate Dehydrogenase | 1.00 | 1.00 | 1.00 |
| Mean Corpuscular Hemoglobin | 0.82 | 0.79 | 0.85 |
| Mean Corpuscular Volume | 1.06 | 1.05 | 1.07 |
| Neutrophil Percentage | 1.01 | 1.01 | 1.02 |
| Platelet Count | 1.00 | 1.00 | 1.00 |
| Red Blood Cell Count | 0.70 | 0.57 | 0.86 |
| Sodium | 1.01 | 1.01 | 1.02 |
| Urea | 1.01 | 1.01 | 1.01 |

**Supplementary Table 1.** Hazard ratios and confidence intervals for the general model addressing case-mortality rates between the unvaccinated and vaccinated populations

#

# Supplementary Table 2A

| **Cox Model Report Over 80 years (Group A)** | | | |
| --- | --- | --- | --- |
| **Variables** | **Hazard Ratio** | **Lower CI (95%)** | **Upper CI (95%)** |
| Vaccinated | 0.80 | 0.76 | 0.84 |
| Sex Male | 0.88 | 0.83 | 0.93 |
| Age | 1.07 | 1.06 | 1.07 |
| Medical Center Type Regional | 0.92 | 0.87 | 0.98 |
| Medical Center Type Specialty | 0.89 | 0.84 | 0.94 |
| ICU Stay | 3.78 | 3.53 | 4.05 |
| Preexisting Condition Active Cancer | 1.33 | 1.26 | 1.41 |
| Preexisting Condition Asthma | 0.90 | 0.83 | 0.97 |
| Preexisting Condition Congestive Heart Failure | 0.93 | 0.87 | 0.98 |
| Preexisting Condition Chronic Liver | 1.10 | 1.01 | 1.19 |
| Preexisting Condition Chronic Kidney Disease | 0.79 | 0.74 | 0.84 |
| Preexisting Condition COPD | 1.04 | 0.99 | 1.10 |
| Preexisting Condition Dementia | 1.01 | 0.95 | 1.07 |
| Preexisting Condition Diabetes | 0.96 | 0.91 | 1.02 |
| Preexisting Condition Hyperlipidemia | 0.89 | 0.84 | 0.93 |
| Preexisting Condition Hypertension | 0.97 | 0.90 | 1.03 |
| Preexisting Condition Ischemic Heart Disease | 1.13 | 1.06 | 1.20 |
| Preexisting Condition Obesity | 0.95 | 0.89 | 1.01 |
| Preexisting Condition Stroke | 0.97 | 0.89 | 1.06 |
| C-Reactive Protein | 1.00 | 1.00 | 1.00 |
| Glucose | 1.00 | 1.00 | 1.00 |
| Hematocrit | 0.99 | 0.99 | 1.00 |
| Hemoglobin | 1.10 | 1.03 | 1.18 |
| Normalized Prothrombin Time - INR | 1.04 | 1.02 | 1.06 |
| Lactate Dehydrogenase | 1.00 | 1.00 | 1.00 |
| Mean Corpuscular Hemoglobin | 0.82 | 0.79 | 0.85 |
| Mean Corpuscular Volume | 1.06 | 1.05 | 1.07 |
| Neutrophil Percentage | 1.01 | 1.01 | 1.02 |
| Platelet Count | 1.00 | 1.00 | 1.00 |
| Red Blood Cell Count | 0.70 | 0.57 | 0.86 |
| Sodium | 1.01 | 1.01 | 1.02 |
| Urea | 1.01 | 1.01 | 1.01 |

**Supplementary Table 2A.** Forest HR analysis group A

# Supplementary Table 2B

| **Cox Model Report Over 70 years (Group B)** | | | |
| --- | --- | --- | --- |
| **Variables** | **Hazard Ratio** | **Lower CI (95%)** | **Upper CI (95%)** |
| Vaccinated | 0.80 | 0.76 | 0.84 |
| Sex Male | 0.88 | 0.83 | 0.93 |
| Age | 1.07 | 1.06 | 1.07 |
| Medical Center Type Regional | 0.92 | 0.87 | 0.98 |
| Medical Center Type Specialty | 0.89 | 0.84 | 0.94 |
| ICU Stay | 3.78 | 3.53 | 4.05 |
| Preexisting Condition Active Cancer | 1.33 | 1.26 | 1.41 |
| Preexisting Condition Asthma | 0.90 | 0.83 | 0.97 |
| Preexisting Condition Congestive Heart Failure | 0.93 | 0.87 | 0.98 |
| Preexisting Condition Chronic Liver | 1.10 | 1.01 | 1.19 |
| Preexisting Condition Chronic Kidney Disease | 0.79 | 0.74 | 0.84 |
| Preexisting Condition COPD | 1.04 | 0.99 | 1.10 |
| Preexisting Condition Dementia | 1.01 | 0.95 | 1.07 |
| Preexisting Condition Diabetes | 0.96 | 0.91 | 1.02 |
| Preexisting Condition Hyperlipidemia | 0.89 | 0.84 | 0.93 |
| Preexisting Condition Hypertension | 0.97 | 0.90 | 1.03 |
| Preexisting Condition Ischemic Heart Disease | 1.13 | 1.06 | 1.20 |
| Preexisting Condition Obesity | 0.95 | 0.89 | 1.01 |
| Preexisting Condition Stroke | 0.97 | 0.89 | 1.06 |
| C-Reactive Protein | 1.00 | 1.00 | 1.00 |
| Glucose | 1.00 | 1.00 | 1.00 |
| Hematocrit | 0.99 | 0.99 | 1.00 |
| Hemoglobin | 1.10 | 1.03 | 1.18 |
| Normalized Prothrombin Time - INR | 1.04 | 1.02 | 1.06 |
| Lactate Dehydrogenase | 1.00 | 1.00 | 1.00 |
| Mean Corpuscular Hemoglobin | 0.82 | 0.79 | 0.85 |
| Mean Corpuscular Volume | 1.06 | 1.05 | 1.07 |
| Neutrophil Percentage | 1.01 | 1.01 | 1.02 |
| Platelet Count | 1.00 | 1.00 | 1.00 |
| Red Blood Cell Count | 0.70 | 0.57 | 0.86 |
| Sodium | 1.01 | 1.01 | 1.02 |
| Urea | 1.01 | 1.01 | 1.01 |

**Supplementary Table 2B:** Forest HR analysis group B

## Supplementary table 2C

| **Cox Model Report Over 60 years (Group C)** | | | |
| --- | --- | --- | --- |
| **Variables** | **Hazard Ratio** | **Lower CI (95%)** | **Upper CI (95%)** |
| Vaccinated | 0.80 | 0.76 | 0.84 |
| Sex Male | 0.88 | 0.83 | 0.93 |
| Age | 1.07 | 1.06 | 1.07 |
| Medical Center Type Regional | 0.92 | 0.87 | 0.98 |
| Medical Center Type Specialty | 0.89 | 0.84 | 0.94 |
| ICU Stay | 3.78 | 3.53 | 4.05 |
| Preexisting Condition Active Cancer | 1.33 | 1.26 | 1.41 |
| Preexisting Condition Asthma | 0.90 | 0.83 | 0.97 |
| Preexisting Condition Congestive Heart Failure | 0.93 | 0.87 | 0.98 |
| Preexisting Condition Chronic Liver | 1.10 | 1.01 | 1.19 |
| Preexisting Condition Chronic Kidney Disease | 0.79 | 0.74 | 0.84 |
| Preexisting Condition COPD | 1.04 | 0.99 | 1.10 |
| Preexisting Condition Dementia | 1.01 | 0.95 | 1.07 |
| Preexisting Condition Diabetes | 0.96 | 0.91 | 1.02 |
| Preexisting Condition Hyperlipidemia | 0.89 | 0.84 | 0.93 |
| Preexisting Condition Hypertension | 0.97 | 0.90 | 1.03 |
| Preexisting Condition Ischemic Heart Disease | 1.13 | 1.06 | 1.20 |
| Preexisting Condition Obesity | 0.95 | 0.89 | 1.01 |
| Preexisting Condition Stroke | 0.97 | 0.89 | 1.06 |
| C-Reactive Protein | 1.00 | 1.00 | 1.00 |
| Glucose | 1.00 | 1.00 | 1.00 |
| Hematocrit | 0.99 | 0.99 | 1.00 |
| Hemoglobin | 1.10 | 1.03 | 1.18 |
| Normalized Prothrombin Time - INR | 1.04 | 1.02 | 1.06 |
| Lactate Dehydrogenase | 1.00 | 1.00 | 1.00 |
| Mean Corpuscular Hemoglobin | 0.82 | 0.79 | 0.85 |
| Mean Corpuscular Volume | 1.06 | 1.05 | 1.07 |
| Neutrophil Percentage | 1.01 | 1.01 | 1.02 |
| Platelet Count | 1.00 | 1.00 | 1.00 |
| Red Blood Cell Count | 0.70 | 0.57 | 0.86 |
| Sodium | 1.01 | 1.01 | 1.02 |
| Urea | 1.01 | 1.01 | 1.01 |

**Supplementary Table 2C:** Forest HR analysis group C

## Supplementary table 2D

| **Cox Model Report Over 40 years (Group D)** | | | |
| --- | --- | --- | --- |
| **Variables** | **Hazard Ratio** | **Lower CI (95%)** | **Upper CI (95%)** |
| Vaccinated | 0.80 | 0.76 | 0.84 |
| Sex Male | 0.88 | 0.83 | 0.93 |
| Age | 1.07 | 1.06 | 1.07 |
| Medical Center Type Regional | 0.92 | 0.87 | 0.98 |
| Medical Center Type Specialty | 0.89 | 0.84 | 0.94 |
| ICU Stay | 3.78 | 3.53 | 4.05 |
| Preexisting Condition Active Cancer | 1.33 | 1.26 | 1.41 |
| Preexisting Condition Asthma | 0.90 | 0.83 | 0.97 |
| Preexisting Condition Congestive Heart Failure | 0.93 | 0.87 | 0.98 |
| Preexisting Condition Chronic Liver | 1.10 | 1.01 | 1.19 |
| Preexisting Condition Chronic Kidney Disease | 0.79 | 0.74 | 0.84 |
| Preexisting Condition COPD | 1.04 | 0.99 | 1.10 |
| Preexisting Condition Dementia | 1.01 | 0.95 | 1.07 |
| Preexisting Condition Diabetes | 0.96 | 0.91 | 1.02 |
| Preexisting Condition Hyperlipidemia | 0.89 | 0.84 | 0.93 |
| Preexisting Condition Hypertension | 0.97 | 0.90 | 1.03 |
| Preexisting Condition Ischemic Heart Disease | 1.13 | 1.06 | 1.20 |
| Preexisting Condition Obesity | 0.95 | 0.89 | 1.01 |
| Preexisting Condition Stroke | 0.97 | 0.89 | 1.06 |
| C-Reactive Protein | 1.00 | 1.00 | 1.00 |
| Glucose | 1.00 | 1.00 | 1.00 |
| Hematocrit | 0.99 | 0.99 | 1.00 |
| Hemoglobin | 1.10 | 1.03 | 1.18 |
| Normalized Prothrombin Time - INR | 1.04 | 1.02 | 1.06 |
| Lactate Dehydrogenase | 1.00 | 1.00 | 1.00 |
| Mean Corpuscular Hemoglobin | 0.82 | 0.79 | 0.85 |
| Mean Corpuscular Volume | 1.06 | 1.05 | 1.07 |
| Neutrophil Percentage | 1.01 | 1.01 | 1.02 |
| Platelet Count | 1.00 | 1.00 | 1.00 |
| Red Blood Cell Count | 0.70 | 0.57 | 0.86 |
| Sodium | 1.01 | 1.01 | 1.02 |
| Urea | 1.01 | 1.01 | 1.01 |

**Supplementary Table 2D:** Forest HR analysis group D

## Supplementary Table 2E

| **Cox Model Report Over 18 years (Group E)** | | | |
| --- | --- | --- | --- |
| **Variables** | **Hazard Ratio** | **Lower CI (95%)** | **Upper CI (95%)** |
| Vaccinated | 0.80 | 0.76 | 0.84 |
| Sex Male | 0.88 | 0.83 | 0.93 |
| Age | 1.07 | 1.06 | 1.07 |
| Medical Center Type Regional | 0.92 | 0.87 | 0.98 |
| Medical Center Type Specialty | 0.89 | 0.84 | 0.94 |
| ICU Stay | 3.78 | 3.53 | 4.05 |
| Preexisting Condition Active Cancer | 1.33 | 1.26 | 1.41 |
| Preexisting Condition Asthma | 0.90 | 0.83 | 0.97 |
| Preexisting Condition Congestive Heart Failure | 0.93 | 0.87 | 0.98 |
| Preexisting Condition Chronic Liver | 1.10 | 1.01 | 1.19 |
| Preexisting Condition Chronic Kidney Disease | 0.79 | 0.74 | 0.84 |
| Preexisting Condition COPD | 1.04 | 0.99 | 1.10 |
| Preexisting Condition Dementia | 1.01 | 0.95 | 1.07 |
| Preexisting Condition Diabetes | 0.96 | 0.91 | 1.02 |
| Preexisting Condition Hyperlipidemia | 0.89 | 0.84 | 0.93 |
| Preexisting Condition Hypertension | 0.97 | 0.90 | 1.03 |
| Preexisting Condition Ischemic Heart Disease | 1.13 | 1.06 | 1.20 |
| Preexisting Condition Obesity | 0.95 | 0.89 | 1.01 |
| Preexisting Condition Stroke | 0.97 | 0.89 | 1.06 |
| C-Reactive Protein | 1.00 | 1.00 | 1.00 |
| Glucose | 1.00 | 1.00 | 1.00 |
| Hematocrit | 0.99 | 0.99 | 1.00 |
| Hemoglobin | 1.10 | 1.03 | 1.18 |
| Normalized Prothrombin Time - INR | 1.04 | 1.02 | 1.06 |
| Lactate Dehydrogenase | 1.00 | 1.00 | 1.00 |
| Mean Corpuscular Hemoglobin | 0.82 | 0.79 | 0.85 |
| Mean Corpuscular Volume | 1.06 | 1.05 | 1.07 |
| Neutrophil Percentage | 1.01 | 1.01 | 1.02 |
| Platelet Count | 1.00 | 1.00 | 1.00 |
| Red Blood Cell Count | 0.70 | 0.57 | 0.86 |
| Sodium | 1.01 | 1.01 | 1.02 |
| Urea | 1.01 | 1.01 | 1.01 |

**Supplementary Table 2E:** Forest HR analysis group E

## Supplementary Table 3

| **Cox Model Report Stratification Unprotected. Protected and Incomplete** | | | |
| --- | --- | --- | --- |
| **Variables** | **Hazard Ratio** | **Lower CI (95%)** | **Upper CI (95%)** |
| Vaccinated | 0.30 | 0.28 | 0.32 |
| Sex Male | 0.33 | 0.30 | 0.36 |
| Age | 0.90 | 0.85 | 0.96 |
| Medical Center Type Regional | 1.05 | 1.05 | 1.05 |
| Medical Center Type Specialty | 0.91 | 0.85 | 0.97 |
| ICU Stay | 0.90 | 0.84 | 0.96 |
| Preexisting Condition Active Cancer | 2.03 | 1.88 | 2.19 |
| Preexisting Condition Asthma | 1.29 | 1.20 | 1.38 |
| Preexisting Condition Congestive Heart Failure | 0.92 | 0.83 | 1.01 |
| Preexisting Condition Chronic Liver | 0.93 | 0.87 | 1.00 |
| Preexisting Condition Chronic Kidney Disease | 0.83 | 0.77 | 0.89 |
| Preexisting Condition COPD | 1.00 | 0.94 | 1.07 |
| Preexisting Condition Dementia | 1.04 | 0.97 | 1.11 |
| Preexisting Condition Diabetes | 1.02 | 0.96 | 1.09 |
| Preexisting Condition Hyperlipidemia | 0.98 | 0.93 | 1.04 |
| Preexisting Condition Hypertension | 1.08 | 1.00 | 1.17 |
| Preexisting Condition Ischemic Heart Disease | 1.08 | 1.01 | 1.17 |
| Preexisting Condition Obesity | 1.08 | 1.01 | 1.16 |
| Preexisting Condition Stroke | 0.90 | 0.81 | 1.00 |
| C-Reactive Protein | 1.00 | 1.00 | 1.00 |
| Glucose | 1.00 | 1.00 | 1.00 |
| Hematocrit | 1.00 | 0.99 | 1.00 |
| Hemoglobin | 1.11 | 1.03 | 1.21 |
| Normalized Prothrombin Time - INR | 1.03 | 1.01 | 1.06 |
| Lactate Dehydrogenase | 1.00 | 1.00 | 1.00 |
| Mean Corpuscular Hemoglobin | 0.86 | 0.82 | 0.89 |
| Mean Corpuscular Volume | 1.04 | 1.03 | 1.05 |
| Neutrophil Percentage | 1.02 | 1.01 | 1.02 |
| Platelet Count | 1.00 | 1.00 | 1.00 |
| Red Blood Cell Count | 0.70 | 0.54 | 0.90 |
| Sodium | 1.01 | 1.00 | 1.01 |
| Urea | 1.00 | 1.00 | 1.00 |

**Supplementary Table 3:** Hazard Ratios Analysis 3

## Supplementary Figure 1

**
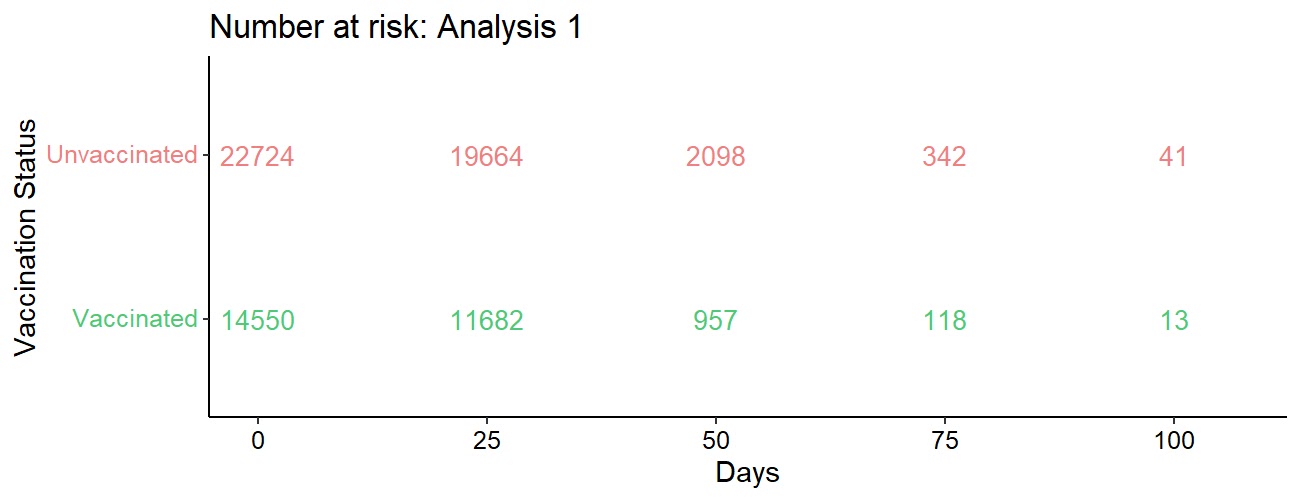
**

**Supplementary Figure 1.** Number of patients at risk at each stage of the analysis.

## Supplementary Figure 2A


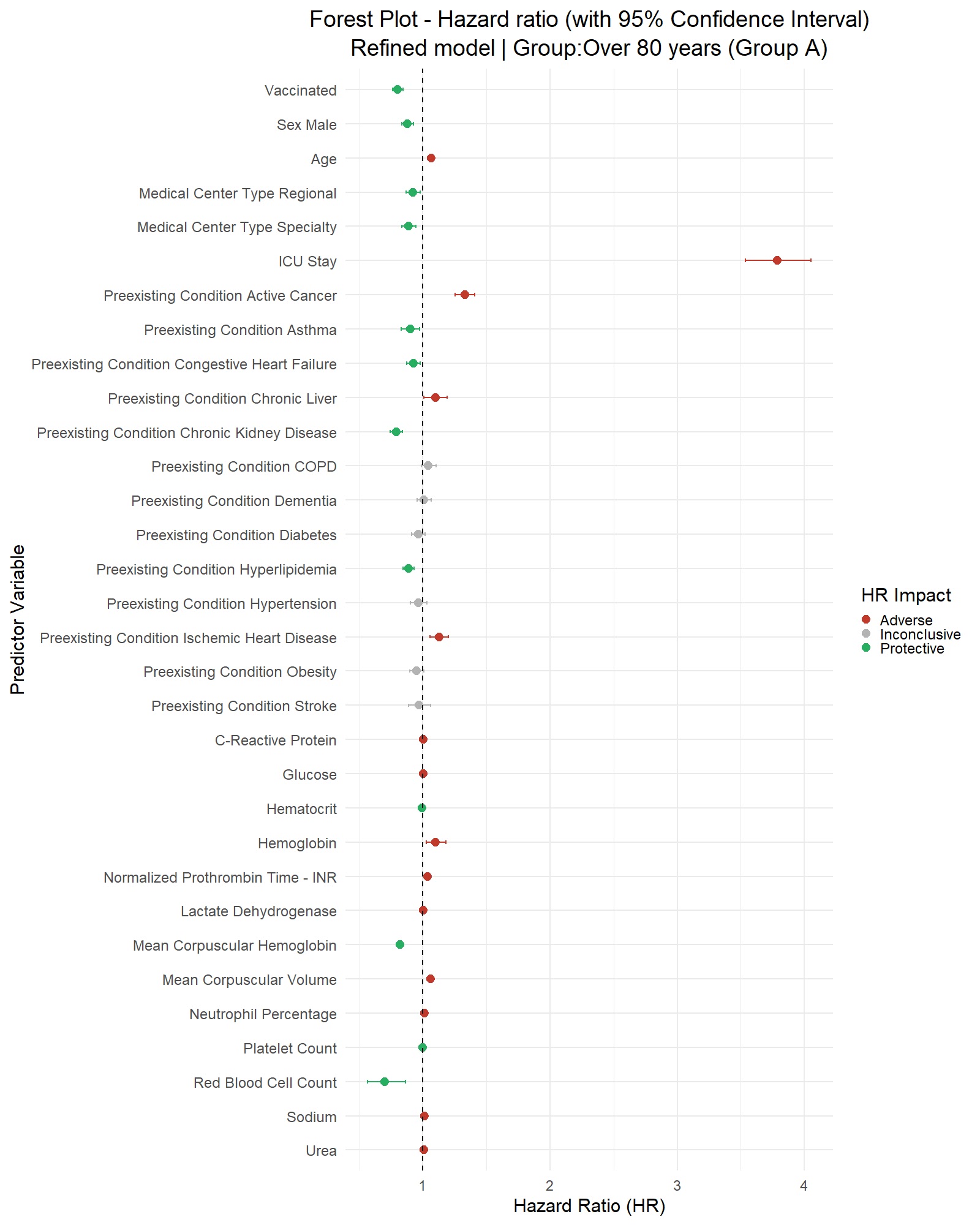


**Supplementary Figure 2A.** Hazard ratios and confidence intervals for models over 80 years addressing case-mortality rates between unvaccinated and vaccinated populations.

## Supplementary Figure 2B


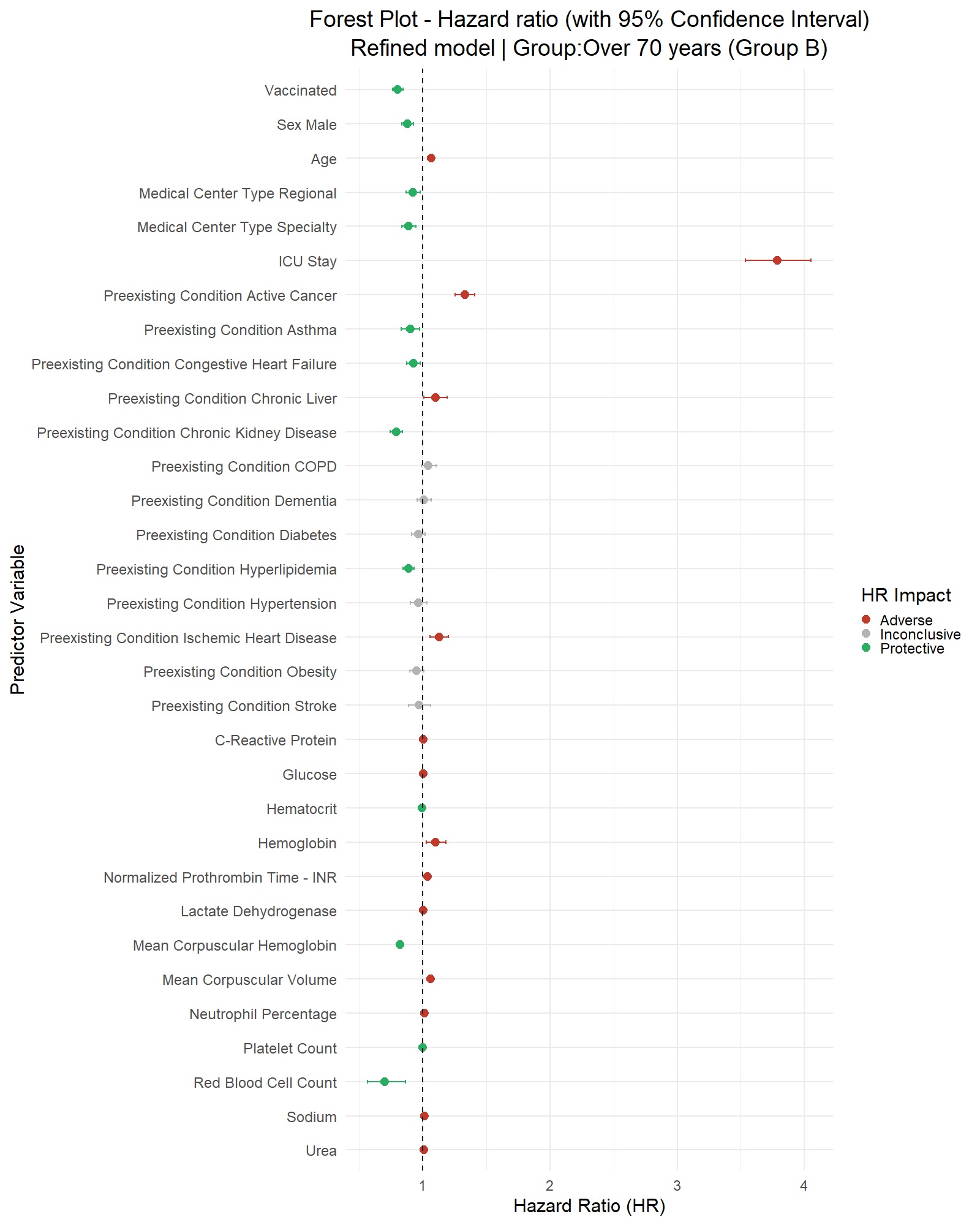


**Supplementary Figure 2B.** Hazard ratios and confidence intervals for models over 70 years addressing case-mortality rates between unvaccinated and vaccinated populations.

## Supplementary Figure 2C


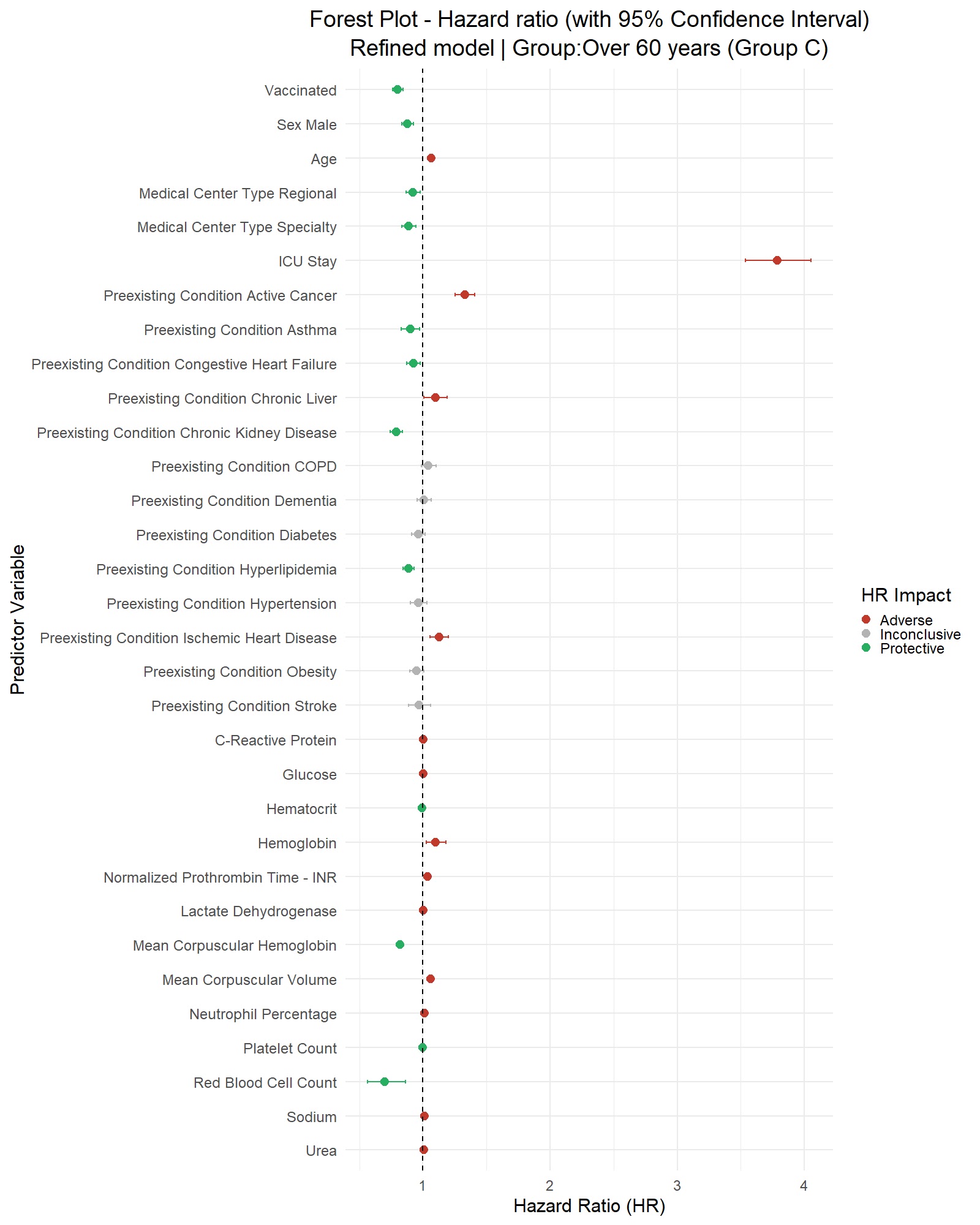


**Supplementary Figure 2C.** Hazard ratios and confidence intervals for models over 60 years addressing case-mortality rates between unvaccinated and vaccinated populations.

## Supplementary Figure 2D


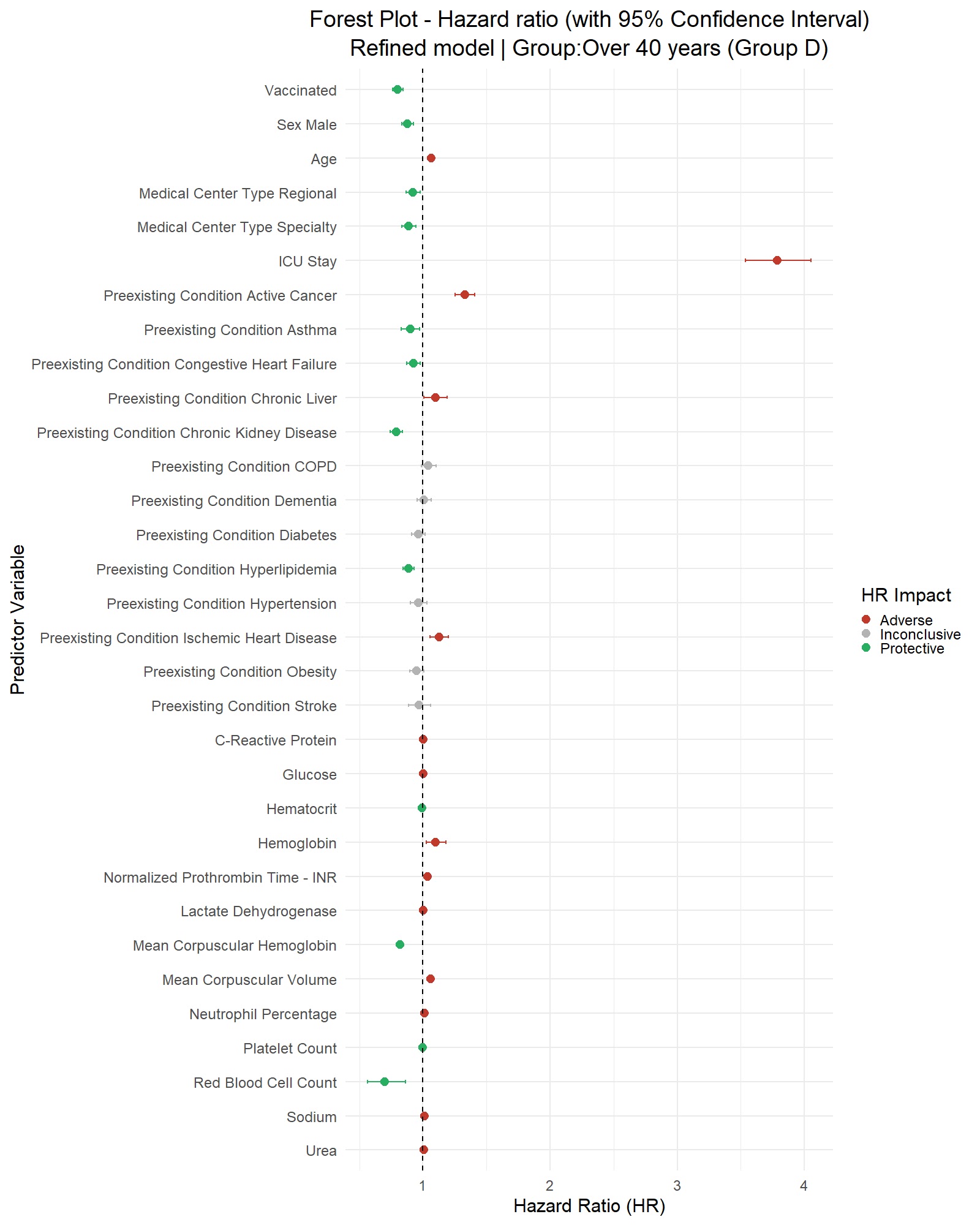


**Supplementary Figure 2D.** Hazard ratios and confidence intervals for models over 40 years addressing case-mortality rates between unvaccinated and vaccinated populations.

## Supplementary Figure 2E


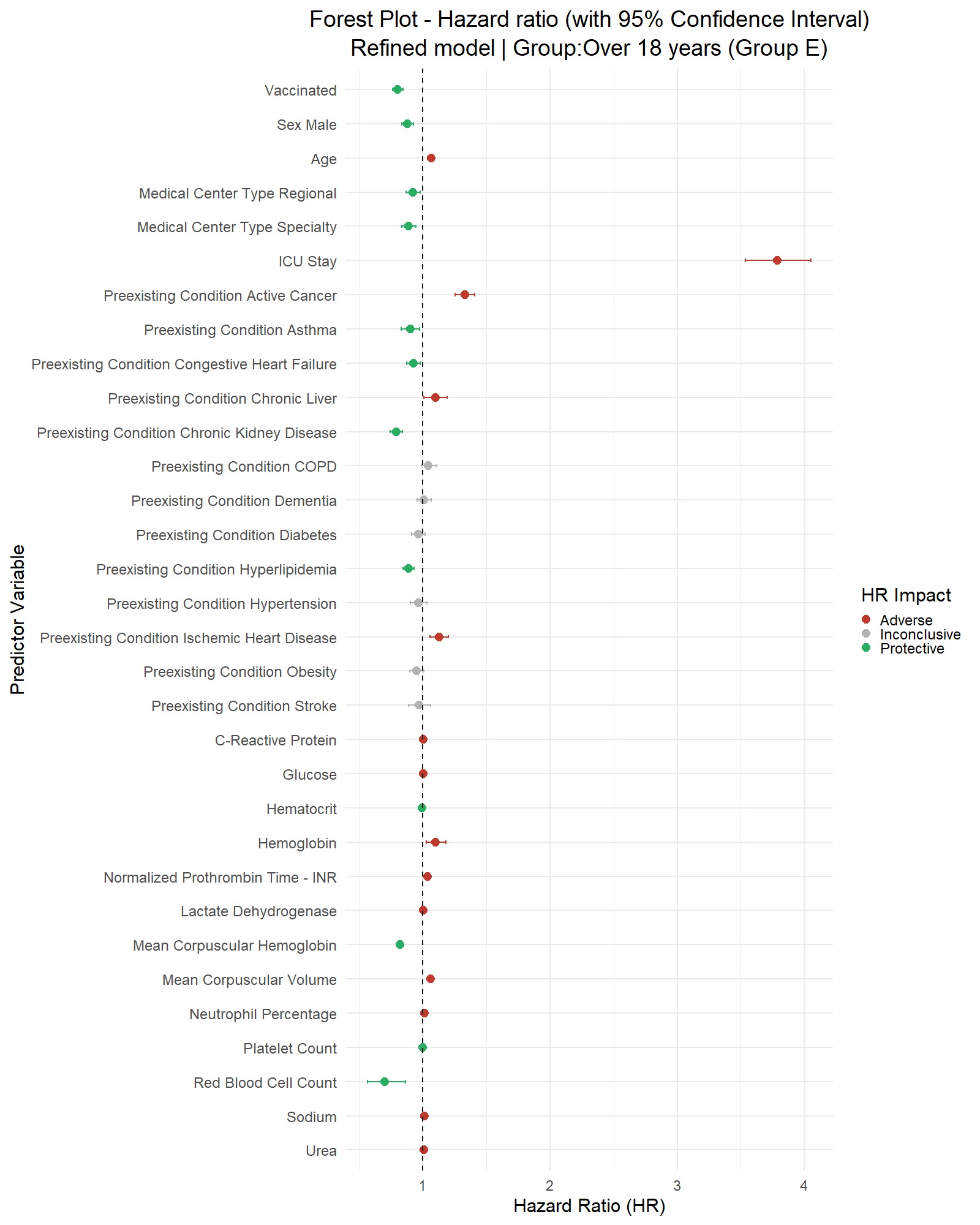


**Supplementary Figure 2E.** Hazard ratios and confidence intervals for models over 18 years addressing case-mortality rates between unvaccinated and vaccinated populations.

# Supplementary Figure 5


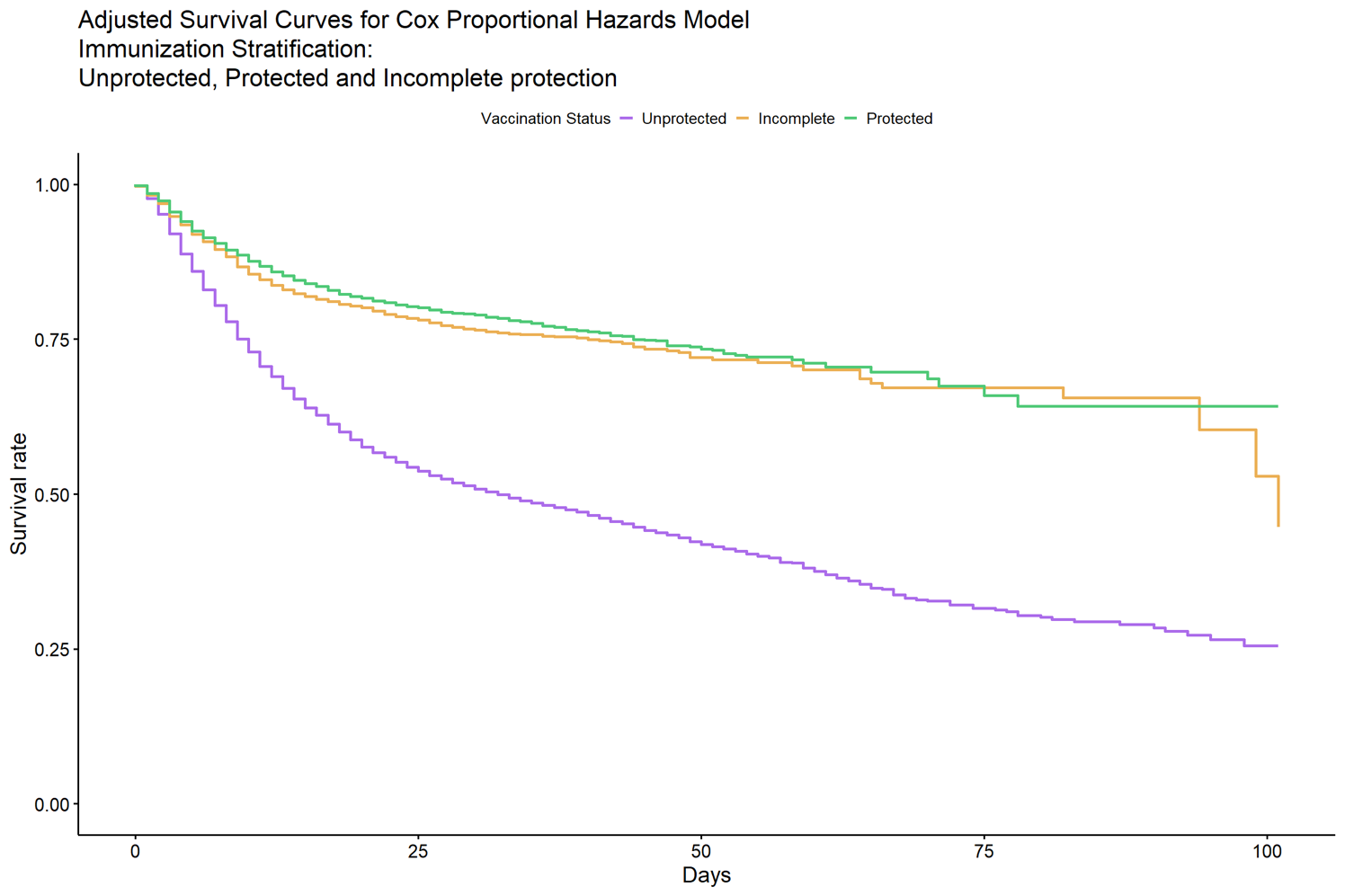


**Supplementary Figure 5.** Adjusted Survival Curves for Cox Proportional Hazards Model for Immunization stratified groups.

# Supplementary Figure 6


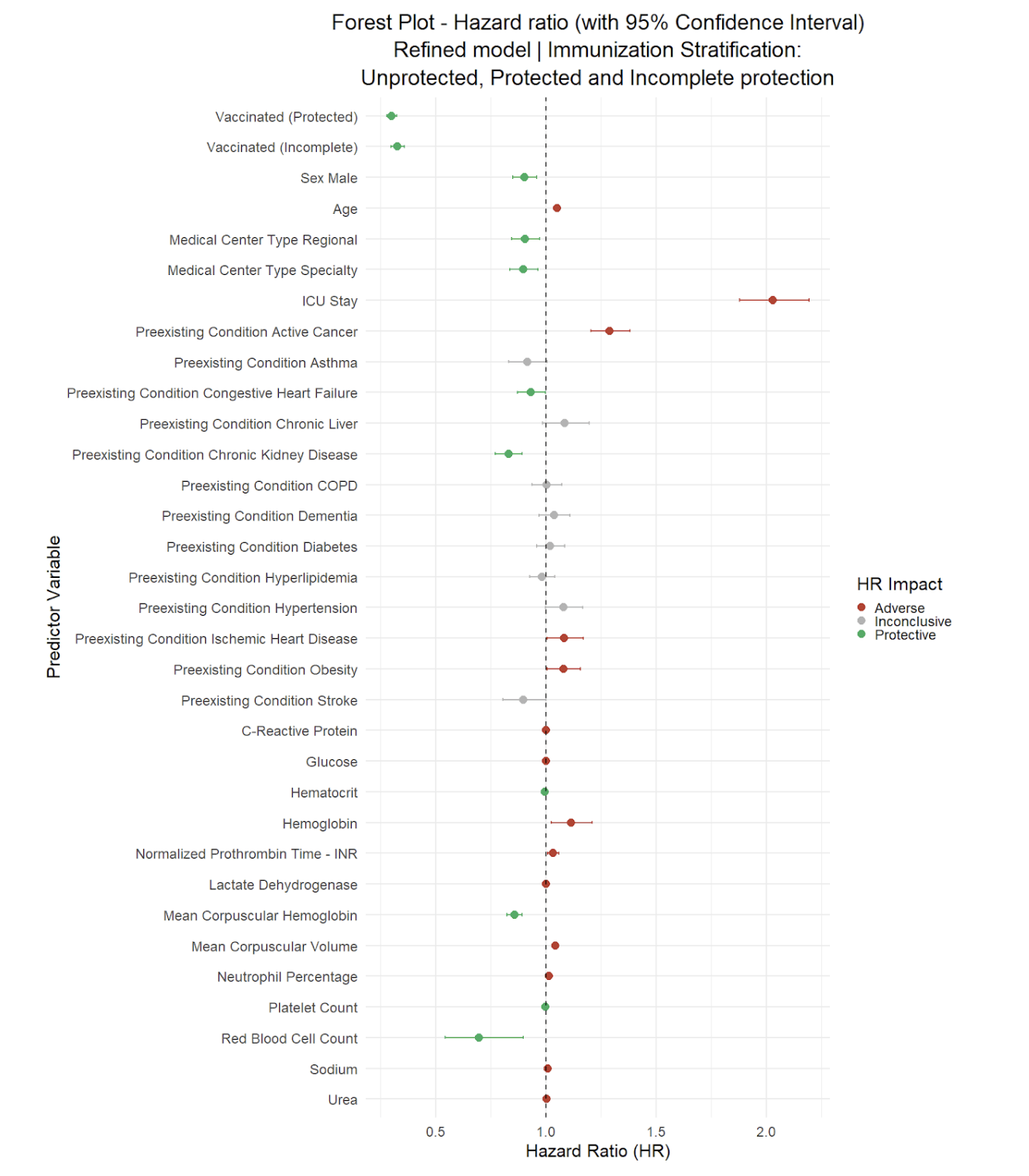


**Supplementary Figure 6.** Hazard Ratios for Immunization stratified groups Forest Plot.

# Supplementary Figure 7


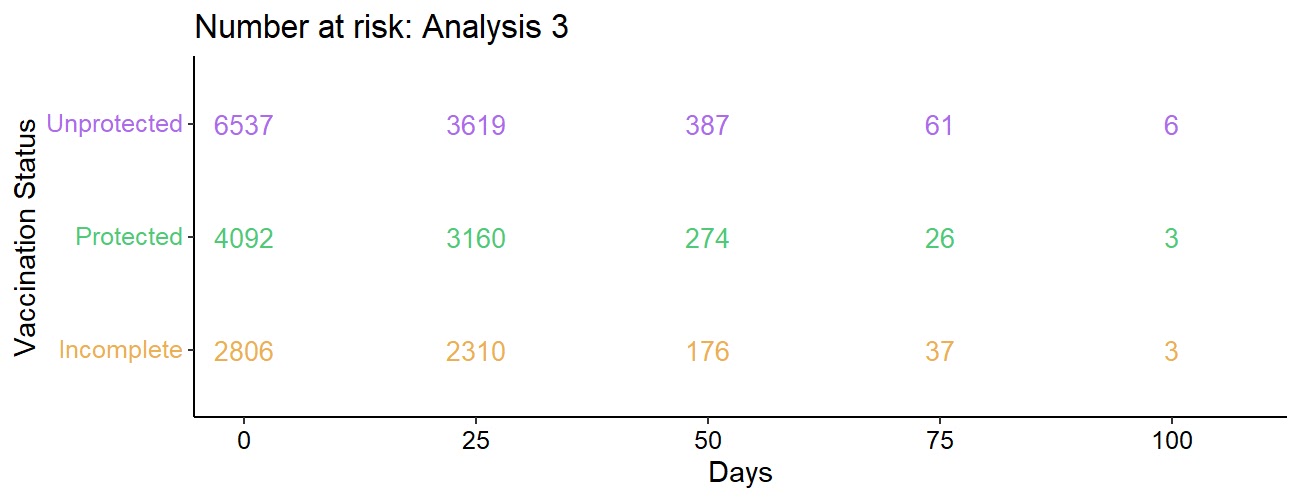


**Supplementary Figure 7.** Number of patients at risk in each stage of the analysis.
